# Virtual Reality for Cardiopulmonary Resuscitation Healthcare Professionals Training: A Systematic Review

**DOI:** 10.1007/s10916-024-02063-1

**Published:** 2024-05-15

**Authors:** Roberto Trevi, Stefania Chiappinotto, Alvisa Palese, Alessandro Galazzi

**Affiliations:** 1 Master Degree in Nursing and Midwifery Science, University of Trieste and Udine, Trieste, Italy; 2 Azienda Sanitaria Universitaria G. Isontina, Trieste, Italy; 3 Department of Medicine, University of Udine, Udine, Italy

**Keywords:** Virtual reality, Healthcare professional, Cardiopulmonary resuscitation, Adult, Child, Neonatal

## Abstract

**Introduction:**

Virtual reality (VR) is becoming increasingly popular to train health-care professionals (HCPs) to acquire and/or maintain cardiopulmonary resuscitation (CPR) basic or advanced skills.

**Aim:**

To understand whether VR in CPR training or retraining courses can have benefits for patients (neonatal, pediatric, and adult), HCPs and health-care organizations as compared to traditional CPR training.

**Methods:**

A systematic review (PROSPERO: CRD42023431768) following the Preferred Reporting Items for Systematic reviews and Meta-Analyses (PRISMA) guidelines. In June 2023, the PubMed, Cochrane Library, Scopus and Cumulative Index to Nursing and Allied Health Literature (CINAHL) databases were searched and included studies evaluated in their methodological quality with Joanna Briggs Institute checklists. Data were narratively summarized.

**Results:**

Fifteen studies published between 2013 and 2023 with overall fair quality were included. No studies investigated patients’ outcomes. At the HCP level, the virtual learning environment was perceived to be engaging, realistic and facilitated the memorization of the procedures; however, limited decision-making, team building, psychological pressure and frenetic environment were underlined as disadvantages. Moreover, a general improvement in performance was reported in the use of the defibrillator and carrying out the chest compressions. At the organizational level, one study performed a cost/benefit evaluation in favor of VR as compared to traditional CPR training.

**Conclusions:**

The use of VR for CPR training and retraining is in an early stage of development. Some benefits at the HCP level are promising. However, more research is needed with standardized approaches to ensure a progressive accumulation of the evidence and inform decisions regarding the best training methodology in this field.

**Supplementary Information:**

The online version contains supplementary material available at 10.1007/s10916-024-02063-1.

## Introduction

New digital technologies have involved every aspect of life with virtual reality (VR) as an innovative tool in training purposes [1]. Although more than 100 definitions have been established to date [2], the term VR identifies various ways of simulating real situations using computers and the aid of specifically developed interfaces [3]. Recently, VR has been included in an overarching term named ‘extended reality’ which includes augmented reality, mixed reality, and other immersive technologies [4]. All these digital technologies have been introduced and tested in different training fields, cardiopulmonary resuscitation (CPR) included, underlining their potentialities in improving and/or maintaining skills [5]. The traditional CPR-standardized face-to-face courses are still offered to provide skills that need complex retraining due to their decreasing over time; however, these traditional courses require physical, human and time resources not easily ensured by the care systems and health-care professionals (HCPs) who are immersed in complex clinical environments, requiring a continuing development of diversified competences and skills [6]. Moreover, when the initial and retraining HCPs learning needs are not provided effectively, there is an increased risk of suboptimal care resulting in poor survival outcomes from cardiac arrest [5]. Considering these issues, recently there has been a call for new CPR training, along with the recommendation for strong research in resuscitation education [7]. In this context, the VR has gained increased space due to its multiple advantages over the traditional face-to-face CPR training methods. Its immersiveness allows participants to completely experience a realistic scenario, developing the skills expected in the real world [8]. VR also ensures involvement and interactivity as compared to the traditional training methods, maximizing the memorization through the feedback received. Moreover, spaces and resources required are limited given that it can be received anywhere, even at home [9]. However, while the use of VR in the basic CPR training of lay adults has been summarized recently regarding its capacity to improve skills [10], no summary of the evidence available concerning VR CPR training among HCPs has been provided to date. Therefore, the intent of this study was to describe the state of the research in this field and detect whether VR CPR training or retraining can have benefits for patients, HCPs and the health-care system.

## Methods

### Study design

A systematic review was performed following the Preferred Reporting Items for Systematic reviews and Meta-Analyses (PRISMA) guidelines [11]. The review protocol was registered in the PROSPERO database (CRD42023431768).

### Research question

The research question was: “Are there any differences in the outcomes between the traditional face-to-face CPR training methods (hereafter, traditional CPR training) and the CPR training based on VR (hereinafter, VR CPR) among HCPs?” Following the Joanna Briggs Institute methodology, the Patient, Intervention, Comparison and Outcome (PICO) framework was used [12]. Specifically, the following were considered: (a) the P as representing the HCPs in any setting; (b) the I as the basic or advanced training for neonatal, pediatric, and adult CPR using VR; (c) the C as the traditional CPR training (e.g., conventional low-fidelity manikin-based training); and d) the O as outcomes measured at the patient (e.g., mortality, neurological), HCP (e.g., satisfaction) and health-care system (e.g., efficiency) levels.

### Inclusion and exclusion criteria

The following studies were eligible for inclusion: randomized controlled trial (RCT), experimental, quasi-experimental and observational primary studies including HCPs (e.g., nurses, physicians, midwives or other); working in any type of setting (e.g., hospital, community, training centers); attending CPR training with VR [13]; and published in English or Italian at any time. Studies including students in a limited proportion compared to the involved HCPs or only in the control group, were eligible. Therefore, studies including lay people or only HCP students; only concerning non-VR CPR training; as well as literature reviews, qualitative studies, letters to editors, protocols, commentaries, and books, were all excluded. The retrieved reviews were inspected manually in their references to identify potential eligible studies.

### Search methods

The search was conducted in PubMed, Cochrane Library, Scopus, and Cumulative Index to Nursing and Allied Health Literature (CINAHL) databases, in June 2023. The keywords (Medical Subject Headings or free text-terms) used were: “Virtual Reality”; “Healthcare Professional”; “Cardiopulmonary Resuscitation”, in combination with the Boolean operators “AND” or “OR”. The search strings are reported in the Supplementary Table 1.

### Study selection

A total of 1042 records were identified, 335 from the databases and 707 from citation searching. First, studies from the databases were screened. Duplicates (n = 78) were eliminated. During the screening process at the title and abstract levels, 213 studies were excluded as not satisfying the inclusion criteria. Subsequently, full texts were read and 31 were not eligible. Four articles did not clearly define the population (e.g., HCPs, lay people or students); therefore, the corresponding authors were contacted and only two answered allowing to exclude the articles according to the inclusion/exclusion criteria; the other two authors did not provide any answer, thus giving the impossibility to assess eligibility of the studies, these were excluded. The inclusion of one study was debated among researchers because it was not made explicit the extended reality used [14]; for this reason, it was excluded.

Articles from citation searching were also screened. From a total of 707 studies, 31 were excluded because duplicates. Of the remained, 676 were excluded because they did not satisfy the inclusion criteria. At the end, two articles were retrieved and included.

**Fig. 1:**
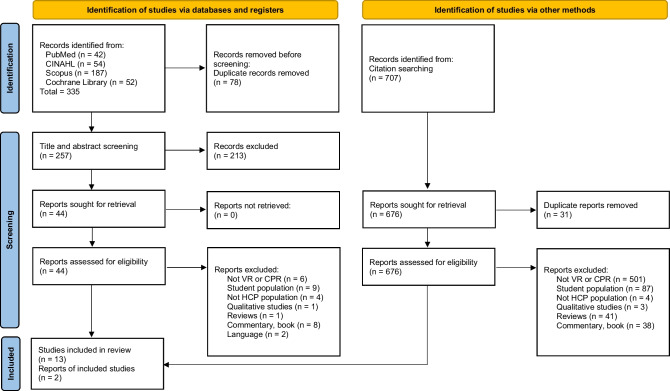
Flow diagram of the study selection process – Preferred Reporting Items for Systematic Reviews and Meta-Analyses (PRISMA) [11] **Legend:** CINAHL, Cumulative Index to Nursing and Allied Health Literature; VR, virtual reality; CPR, cardio-pulmonary resuscitation; HCP, health-care professional; n, number

Overall, a total of 15 studies were considered (Fig. 1). The entire selection process involved two researchers (RT, AG) who worked independently; in case of disagreement a third researcher (SC) was consulted to reach consensus.

### Quality assessment

The Joanna Briggs Institute checklists for randomized controlled trials [15], quasi-experimental [16], and cross-sectional studies [17], respectively were used. Some studies did not clearly indicate the design [18–20]: in the case of pre-post measures (even with a randomized sample), studies were considered quasi-experimental, while in the case of a single point evaluation, these were considered cross-sectional. The assessment was performed independently by two researchers (RT, SC); in the case of disagreement a third researcher (AG) was consulted to reach consensus.

### Data extraction and synthesis

A data extraction table was firstly piloted in three studies and no modifications were required. Then, the data extraction process was conducted by two researchers (RT, AG) populating the grid with the following data: author(s), publication year, country where the study has been conducted; study objective and design; type of CPR (basic or advanced), training or retraining; data collection year and duration; sampling, sample, setting; virtual simulation modality, devices; main features of the CPR training offered as comparator; tools for outcome measurement; and main findings. A third researcher (AP) verified this process and resolved disagreements. Then, studies were grouped according to the CPR target population. A narrative synthesis of their main characteristics and quality was first provided [21]; then, the VR CPR training and comparison modes were described in their main features; a summary of the outcomes measured, and their metrics were then provided.

## Results

### Study characteristics

There were included 15 studies (Table 1) published between 2013 and 2023 and conducted in Europe [18, 19, 27–29], in North America [20, 24, 25, 31], in Africa [32, 33], in Asia [26, 30], and in Central America [23]; one study did not report the country [22].

**Table 1:** Characteristics of the included studies

**Author** **Publication year** **Country**	**Objective** **Design** **Basic/advanced CPR** **Training/retraining** **Data collection year, duration**	**Sampling** **Sample** **Setting**	**Virtual simulation modality** **Devices**	**Comparison**	**Tools for outcome measurement**	**Main results**
***Adults***						
Brzozowski et al. [22] 2022 ND	To investigate the feasibility of using virtual simulation in CPR training Pre–post study Basic CPR Training ND, three months	Convenience sampling 4 RNs and 3 nursing assistants on voluntary basis, during their free time Age: ND Female: ND Professional experience: from less than 1 year to more than 5 years 2 adult, acute care, medical-surgical units at a Level I trauma centre	Virtual online simulations of CPR offered via online video conferencing technology The process of all the simulations required less than 25 min Sessions were held for a period of 1 week each month for 3 consecutive months. Between 48 and 79 time slots were available weekly 20-s presentation video followed by the scenario. This is followed by a 1-min video, a mutual feedback debriefing with the researchers and again a scenario	Single group pre–post test	Prior to the baseline virtual simulation, demographic and self-report confidence surveys were completed online by participants The baseline assessment checklist was completed by the project team during each virtual simulation. During the repeated simulation, the project team completed the same checklist, and participants completed the self-confidence survey Self-confidence was measured with a tool minimally adapted from a previous study, evaluating 16 activities to be done in the first five minutes waiting for the ALS team, Likert scale from 1 (strongly disagree) to 4 (strongly agree) Initiation times for various CPR procedures (baseline virtual simulation vs second virtual simulation)	Participants’ confidence scores improved from baseline after VR; they were confident in: - identifying an unresponsive and/or deteriorating patient (check for a pulse, assess for breathing) + 14.3% - managing a patient in crisis prior to the rapid response team or code team arrival + 14.3% - using the defibrillator on their unit (applying pads, charging, shocking) + 28.6% - participating in a group simulation + 28.6% - giving SBAR report to the rapid response/code team + 14.3% Participants maintained their confidence in the remaining six items Average resuscitation response times were all reduced from baseline to immediate post VR simulation The average time decrease was - rhythm analysis 90.3 s - defibrillation 87.3 s
Buttussi et al. [18] 2013 Udine, Italy	To assess the effectiveness, the gain in knowledge and decision-making skills of a 3D serious game for ALS retraining The perceived effectiveness of the serious game and ALS providers’ willingness to continue retraining with it Experimental study Advanced CPR ND 2011, three months	Convenience sampling 40 participants who had already followed an ALS training course, physicians and nurses, ALS providers, on a voluntary basis, and rewarded with 6 medical education credits for attending Age: 35.5 (SD 6.4) years Female: 25 (62.5%) 70% worked in the emergency area and only 15% used a video game University classroom	EMSAVE, a single-player serious game using a 3D scenario-based ALS simulation for computers The entire simulation process required 1 h 4 retraining sessions took place with 10 participants per session 15-min tutorial showing how EMSAVE worked, with the opportunity to ask questions Participants played a cardiac arrest scenario to familiarize themselves with it Participants played 2 retraining scenarios with 2 different causes (anaphylactic shock and ventricular tachycardia) of cardiac arrests set in different virtual environments (living room and train station) EMSAVE provided self-correction during playing Participants use desktop computers and earphones	Single group pre-post-test	The ALS test was created ad hoc and composed by 38 questions Baseline questionnaire (demographics, computer use and general ALS information) and regarding ALS knowledge and skills with a multiple-choice test (pre-test) The ALS test was administered after VR experience (post-test) Participants were administered a final 13-item questionnaire (Likert scale) to assess the perceived validity, effectiveness, willingness to use, usability, engagement and overall appreciation of the EMSAVE The ALS test was administered after 3 months (retention test) via email	All participants except one evaluated EMSAVE as a valuable tool to refresh ALS knowledge and skills Considering frequency of training with the 3D serious game, 75% of participants thought they should use it for training at least once every 6 months (15% even once a week or more) There was a large agreement on all the positive statements about EMSAVE and the training experience: all but one participant considered EMSAVE as a valid ALS self-training tool and more than 75% of them thought it increased their confidence in ALS procedures The proposed scenarios were considered realistic by 80% of participants After using EMSAVE, the number of correct answers increased by 4.8 (95% CI + 3.4, + 6.2, p < 0.001) and all but one participant improved After 3 months (39/40 participants), despite an expected decrease in ALS knowledge and skills (− 1.9 correct answers, 95% CI − 0.6, − 3.3, p < 0.01), there was a significant retention benefit (+ 2.9 correct answers per participant, 95% CI + 1.5, + 4.2, p < 0.001) The number of correct answers in the post-test increased in 79% of questions
García Fierros et al. [23] 2021 Mexico City, Mexico	To measure the CPR performance on training manikins by using a sensor that records the strength of the compressions applied by the user during the session Experimental study Basic CPR ND ND	Convenience sampling 4 participants: 2 not trained users in the basic technique of CPR and 2 trained users Age: ND Female: ND 2 trained users were practitioners and trainers in first-aid techniques Superior School of Mechanical and Electrical Engineering of the Instituto Polytechnic Nacional	Virtual CPR is a mobile VR application that implemented an interactive virtual scenario using VR lenses A real manikin and a sensor on its chest are required The main interactive elements are: 1. the user interface, composed of panels, images, sounds and buttons that help to control the simulation and decide whether to take an action 2. a three-dimensional manikin that simulates an individual requiring CPR 3. an interface to generate animations displayed by the user, helping to perceive the actions in the simulation 4. a realistic environment of a university classroom	ND	32 tests A multifactorial analysis of variance including four factors for a CPR session: previous CPR training, frequency of compressions, presence of auditory suggestions and presence of colour indicator	The more previous training in CPR a user of the virtual CPR system has, the greater the percentage of correct compressions obtained from a virtual CPR session Setting the rate to 100 or 150 compressions per minute, turning on or off the auditory suggestions and turning the colour indicator on or off during the session had no significant effect on the results Previous user training in CPR techniques has a significant and positive effect on the percentage of correct compressions achieved through a virtual CPR session (F-Ratio 14.95; p = 0.0006)
Katz et al. [24] 2020 New York, USA	To explore the utility of a voice-based VR ACLS team leader refresher as compared with HFS Cross-sectional study Advanced CPR Retraining ND	Convenience sampling 25 postgraduate year 2 anaesthesiology residents Age: 25 to 35 Female: 8 (32%) 1 year past their first ACLS certification; with the same rotations and passed the required examinations Fewer participants were familiar with VR compared with HFS (36% vs 100%; p < 0.001) Mount Sinai Human Emulation Education and Evaluation Lab for Patient Safety and Professional Study Centre at the Icahn School of Medicine	An educational module testing participants on the same ALS algorithms Sessions were run on laptops and VR headsets Participant were team leader of a critical patient in a radiology suite The VR intervention used voice controls, with a virtual team to which the participant delegated ACLS tasks The HFS intervention was managed by ACLS instructors (anaesthesiologists) A human patient simulator manikin was used with MUSE software Participants could only give vocal commands to the team about ACLS procedures	Participants were randomized to HFS or VR training and then crossed after a 2-week of washout Participants and proctors were not informed about the study aims	List of ACLS tasks: incorrect answers or answers given after 15 s (except for chest compressions, which were required to be within 10 s) were graded as incorrect At the end of sessions each day, proctors were asked to fill out a NASA-TLX form indicating their performance and experience throughout the day. This is a validated instrument for measuring perceived workloads for performing tasks, graded on a 20-point scale in six domains Instructors were given a rubric of all current ACLS algorithms to be tested along with a rubric against which to grade in a Correct, Correct with Assistance, and Incorrect manner Cost analysis (purchasing orders for equipment and salaries for personnel involved)	Self-reported satisfaction and utilization scores were similar; significantly more participants felt HFS provides better feedback: 99 (IQR 89–100) vs 79 (IQR 71–88); p < 0.001 Technical scores were higher in the HFS group (72.7, IQR 60.0–78.2 vs 47.0, IQR 40.0–58.0; p < 0.001); however, non-technical scores for decision-making and communication were not significantly different between modalities VR sessions were 21 (IQR 19–24) min shorter than HFS sessions The National Aeronautics and Space Administration task load index scores for proctors were lower in each category (mental demand, physical demand, temporal demand, performance impact, effort, frustration), and VR sessions were estimated to be USA $103.68 less expensive in a single-learner, single-session model
Khanal et al. [25] 2014 Phoenix, Arizona, USA	To investigate the efficacy of using a VR-based simulator intended for team training in ACLS Randomized controlled trial Advanced CPR Training ND	Convenience sampling 148 ACLS-certified clinicians divided into 26 teams Aged: ND Female: 138 (93.2%) Banner Health Simulation Education and Training Centre	VR simulator based on MySQL database management system with four different levels (role, interface, feedback and server), six diversified roles and three interface components (visual, auditory and touch) using systems called Team Speak and Noving Falcon Each team was randomly assigned to one of the three treatment groups: control (traditional ACLS training – low-fidelity manikin), persuasive (VR ACLS training with comprehensive feedback components), or minimally persuasive (VR ACLS training with limited feedback components) Participants were randomly assigned to one of the six ACLS roles: compressor, medicator, defibrillator, airway manager, respirator and leader. Each role was associated with performing a specific set of tasks The study phases were 1) administration of a demographic questionnaire 2) pre-test to assess baseline 3) didactic training (25-min presentation of ACLS protocols with audio recording) 4) intervention groups 5) post-test evaluation 6) final questionnaire about the experience For VR experience, the feedback module involves the task of providing visual (including textual) and auditory feedback to the users during and after a training session	The teams were tested across two different ACLS scenarios (VF/VT without pulse, and PEA)	An electronic checklist (items as primarily tasks that correspond to AHA guidelines for ACLS) was developed and validated internally by a team of expert ACLS trainers, who evaluated participants’ performance Participants were asked to answer a questionnaire regarding the training experience	The difference in performance between control and persuasive groups was not statistically significant; differences in performance between control and minimally persuasive groups was significant (p = 0.05 for PEA and p = 0.02 for VF/VT) The pre–post comparison of performances of the groups showed that control (p = 0.017 for PEA, p = 0.01 for VF/VT) and persuasive (p = 0.02 for PEA, p = 0.048 for VF/VT) groups improved their performances significantly, whereas the minimally persuasive group did not (p = 0.45 for PEA, p = 0.46 for VF/VT)
Lee et al. [26] 2022 Korea	To describe the development of an XR for BLS simulator, how it works and its evaluation by experts Cross-sectional study Basic CPR ND ND	Convenience sampling 16 experts (9 emergency medicine specialists and 7 emergency medical technicians), 6 different institutions Age: ND Female: ND BLS education experience: 5.6 (SD 4.4) years ND	The XR-BLS simulator was created by employing a half-torso manikin in a virtual reality environment and using BLS guidelines A head-mounted display and hand-tracking device were used to perform chest compressions and ventilation and to enable the use of an AED in a virtual environment The primary input for learners was via the use of their own hands, head and voice Hand-tracking technology (Leap Motion tracker) allowed learners to be able to see 3D graphics of their real hands in action and to manipulate 3D objects (i.e., the AED) The manikin (Nurugo B100) was equipped with five sensors for vibration detection, depth and rate of the chest compression, chest compression location, opening of the airway and volume of the rescue breath The AI instructor was programmed to provide real-time feedback (e.g., quality of ventilations or chest compressions) The virtual characters had real human features Participants performed a 20-min learning mode with an AI instructor. Then, the training was performed in the practice mode, in accordance with real-life usage, followed by test mode	Not performed	After the usability test, an expert group survey was conducted with 8 items: 3 about the ease of use of XR-BLS, 3 about the delivery of training, and 2 about the AI instructor	The evaluation of the XR-BLS simulator (joining the options strongly agree and agree) was: - learning to operate the system is easy (100%) - the possibility to see hands and manikin well in VR (100%) - the personal interaction with the system clear and understandable (100%) - to become proficient on BLS using this system (100%) - the information was effective to complete the task and the scenario (100%) - the instruction in VR tutorial were organized, clear and easy to understand (93.8%) - it was easy to understand the AI instructor’s explanations and instructions (100%) - the interaction with the AI instructor is clear and understandable (87.5%)
Peek et al. [27] 2023 Rotterdam, Netherlands	To show that participants who undergo VR training can progress through the CSU-ALS algorithm accurately and as quickly as participants who undertake the traditional training, demonstrating the utility of the CPVR-sim as a replacement for current training protocols Single-blinded randomized controlled trial Advanced CPR Training 2022 May 19th	Stratified block randomization with a block size of 4, distributed according to work experience using a binary cut-off at 4 years of work experience Participants and study personnel were not blinded 28 cardiothoracic surgery residents with: at least 1 year of experience in cardiothoracic surgery, 7% never experienced with CPR after cardiac surgery, 7% had never experienced gaming, 14% never experienced simulation training, 14% never experienced with digital training Age: 31.3 (SD 1.9) years for the VR group and 32.3 (SD 2.4) years for control group Female: ND Department of Cardiothoracic Surgery, University	Participants being randomized to a VR CSU-ALS simulator training arm or a conventional classroom CSU-ALS training arm All participants studied the materials provided before starting the course (guidelines for resuscitation after cardiac surgery, a video including different scenarios for cardiac arrest after cardiac surgery, and a video of a resternotomy procedure) Participants filled in a baseline questionnaire about their work experience, cardiac arrest after cardiac surgery, and emergency resternotomies Participants in the VR group first received a 5-min introductory briefing on how the VR headset and controllers worked Next, they performed the CPVR-sim simulation training, including three different cardiac arrest cases The CPVR-sim took approximately 30–45 min per participant	Participants in the control group received conventional CSU-ALS training from a certified CSU-ALS instructor The training included a presentation of the protocol (15 min) and simulation training (45 min) with a resternotomy manikin	Both initial trainings (control and VR group) were completed with a standardized physical assessment (role play/moulage), using a resternotomy manikin The assessment case was the same to facilitate a direct comparison of participants’ performance After the assessments, the groups were switched Finally, the usefulness, satisfaction and ease of use of VR training was assessed via a questionnaire For the primary outcome, all participants were timed, and the time was recorded at two points: (1) when the team administered the third of three shocks as instructed by the team leader; and (2) when resternotomy was performed as randomly assigned to perform	43% of participants were able to administer stacked shocks within 1 min after conventional training, whereas none of the participants in the VR group reached this time target The mean time to deliver the stacked shocks was 62.7 (SD 8.9) seconds for the control and 85.4 (SD 17.1) seconds for VR groups The resternotomy time target (< 5 min) was reached in 100% of the procedures in the control and in 83% of the procedures in the VR group, in 211.0 (SD 25.5) s for the control group and in 273.0 (SD 21.0) s for VR group In the VR group, the total amount of mistakes (e.g., call resus team, ventilation, removal of steel wires) was lower than that in the control group (11 vs 15 mistakes). However, the number of mistakes per patient case were comparable for both groups: control group 2 (0.8–3.3) versus VR group 2 (1–2) Most participants (76%) learned a lot from the CPVR-sim, and the simulation helped most (72%) of the participants remember the steps required in a CPR situation. Most enjoyed the CPVR-sim (86%), and using VR simulations for learning purposes (86%) The software was easy to use (72%) and quick to learn (93%), without needing written instructions (79%)
Rodríguez-Matesanz et al. [19] 2022 Madrid, Spain	To highlight the limitations of traditional methods using mechanical manikins and the benefits of the new approaches that involve the students in virtual, immersive, and dynamic environments Cross-sectional study Basic CPR ND ND	Convenience sampling 33 participants: 15 HCPs, 10 medical students, 6 professors and 2 traditional objective structured clinical exam examiners, from different nationalities 45.5% participants never tested a VR application before Age: 19 to 57 years Female: ND Hospital	CPR-OSCE VR is a software application that can be executed both on PC or with Oculus Quest It featured multiple scripts to visualize the different environment props and characters, track the user’s location, movement and interaction, and measure the accuracy of the CPR performance The system can be used both with and without an external physical manikin and the extra tracking devices but in this study they were used Performance metrics are generated during the simulation. The user is assisted by an external manikin that provides a point of reference in the simulation, as well as tactile feedback	Not performed	All participants played the same scenario Sensors for automatic detection of participant performance After the simulation a questionnaire was administrated, and regarded: (1) personal data; (2) previous experience with VR, similar technologies, and objective structured clinical exam; (3) user experience with CPR-OSCE VR; and (4) fidelity of CPR-OSCE VR	88% of the participants obtained a correct compression rate, and 65% a positive outcome in the appropriated compression depth applied to the manikin A good level of realism was present for 97% of the participants
Sadeghi et al. [28] 2022 Netherlands	To assess the feasibility and to establish the face and content validity of two clinical scenarios (shockable and not shockable CA) of the CPVR-sim Cross-sectional study Advanced CPR ND ND	Convenience sampling 41 participants: 14 cardiothoracic surgeons, 5 residents, 12 junior physicians, 6 nurse practitioners, 4 medical students. 27% had no experience with emergency resternotomy, 32% had no experience with VR and 17% had no experience with simulation training Age: 43 (IQR 38–55.5) for expert and 30 (IQR 30–42.5) for novice group Female: 14 (34%) Cardiothoracic Surgery Department	The simulation was designed by a multidisciplinary team from different countries An Oculus Quest 2 head-mounted display was used, in combination with two VR controllers and a high-performance laptop Before the simulation, each participant was given a short briefing on the scenario, the use of the VR, and how to interact with the controls and software The team leader could assign tasks to the other participants or run them independently; he/she instructed the virtual colleagues by choosing between different menu options with the joystick When the correct command was given, it was followed by visual and auditory feedback of the instruction	Staff cardiothoracic surgeons and certified CPR training instructors were categorized as expert, while the remaining participants as novices (junior physicians, nurse practitioners, surgical residents, and medical students)	A questionnaire regarding experience with emergency resternotomy, gaming, and VR Scores based on a 5-point Likert scale (1 fully disagree; 5 fully agree) for usefulness, satisfaction, ease of use, effectiveness, and immersiveness. Open questions to assess the advantages and disadvantages of the simulation Face validity was assessed with the questionnaire results on the ease of use, effectiveness and immersiveness of all participants Content validity was assessed by accessing the results (usefulness and satisfaction) of the expert group that performed the PEA scenario	80% reported that it was easy to learn how to interact with the software All experts in the PEA scenario agreed that this VR training method is useful as a supplement to conventional training methods, and 60% agreed it was useful as a supplement to digital training 14% of experts would prefer VR training instead of conventional training; however, 47% of them would prefer VR training instead of digital training 93% of participants reported that the CPVR-sim was a useful method to train infrequently occurring CPR cases after cardiac surgery The most reported advantages were the broad applicability of VR simulation in various CPR scenarios; the possibility of repetitive, personal and quick practice sessions without being restricted by logistical challenges; and that the CPVR-sim is a beneficial method for step-by-step sequence training. Many participants felt it was a fun way of learning The most disadvantages of the current CPVR-sim version were the limited freedom of decision-making, the lack of team training and interaction with a team, and the absence in the CPVR-sim of the pressure and hectic environment during such an emergency, which occasionally made it feel artificial
Semeraro et al. [29] 2013 Bologna, Pisa, Italy	To evaluate a CPR feedback system—Mini-VREM—designed to improve chest compressions during training, in terms of feasibility, effectiveness and acceptance by learners Randomized crossover pilot study Basic CPR ND 2011, November/December	Randomization in 2 groups: with Mini-VREM feedback (FB) or without (NFB) 80 participants divided into 2 groups: nurses and physicians (50% CPR experts) and engineers, students and researchers (50% non-CPR experts) Age: 33.4 (SD 8.22) years Female: 40% At the ICU Department of the Maggiore Hospital and PERCRO Laboratory	Mini-VREM connected to a classic dummy with a sensor (Kinect) positioned in front of the person, connected to a computer equipped with software to give live feedback on the quality of the CPR Participants received an explanation of the Mini-VREM feedback features using a standard video demonstration. All subjects performed a 2-min CC trial, 60-min pause and a second 2-min CC trial The first group (FB/NFB, n = 40) performed CC with Mini-VREM feedback followed by CC without feedback The second group (NFB/FB, n = 40) performed vice versa	The scenario was the same for the study groups, with visual and audio feedback disabled in the no feedback sessions	Comparison by analysing Mini-VREM data downloaded for CC quality evaluation, and administering two questions (answers on a 10 cm visual analogue scale) for effectiveness and exhaustion, and five questions with a 7-point Likert scale (1 = completely disagree, 7 = completely agree) to explore perceptions	The percentage of adequate CCs in the FB group (35.78%) was almost five times higher than in the NFB group (7.27%, p < 0.001) Correct CC rate was achieved in 31.42% of CCs without feedback vs. 72.04% with Mini-VREM (p < 0.001) Correct CC depth was achieved in 24.87% of CCs without feedback vs 47.34% with Mini-VREM (p = 0.002) Compared to no feedback, performing CCs with Mini-VREM guidance was perceived to be more effective (FB 7 (5–8) vs NFB 6 (5–7); p < 0.007); Mini-VREM guidance was perceived to affect fatigue (FB 7 (6–8.88) vs NFB 7 (5–8); p = 0.002) The 7-point Likert scale showed that: - It was difficult to use Mini-VREM 1.5 (SD 1.1) - The numbers and bars on the monitor were easily visible 6.7 (SD 0.8) - The metronome was easily audible during the CPR 6.8 (SD 0.5) - Mini-VREM helped you to perform chest compressions at a rate of 100–120 bpm 6.7 (SD 0.7) - Mini-VREM helped you to perform chest compressions at a depth of 50–60 mm 6.3 (SD 1.1)
Vankipuram et al. [20] 2014 Phoenix, USA	To present the details of the framework and the development methodology associated with a VR-based training simulator for ACLS, a time-critical, team-based medical scenario To report the key findings of a usability study conducted to assess the efficacy of VR simulator Cross-sectional study Advanced CPR Training ND	Randomization 96 ACLS-certified clinicians Age: ND Female: ND Simulation education and Training Centre at Banner Good Samaritan Medical	The primary input devices for all users were the mouse and keyboard. The setting for the VR-based training was a standard hospital room All participants were organized into 16 different teams; each consisting of 6 randomly assigned members to represent 6 ACLS key roles (leader, medicator, defibrillator, airway manager, respirator, and compressor) The participants were initially asked to view a 20-min tutorial video specific to each role This was followed by a 30-min session where they were asked to run through four to five virtual mock codes each approximately 5 min in length After the session, the participants were required to fill out a questionnaire about the ease of use and system usability	The 16 teams were randomly further split into 2 groups that interacted with 2 different versions of the VR simulator (one having all persuasive features and the other with a limited set of persuasive features) Persuasive feature was provided in the form of sounds, animations, and text messages that flash across the centre of the screen for 3 s	The questionnaire was composed of 6 questions, on a Likert scale of 1–5 (1-strongly negative, 5-strongly positive)	The ratings for the minimally persuasive group exceeded those for the persuasive group for both ease of use (p = 0.0813) and usability (p = 0.0944) This was probably due to fewer interruptions in the persuasive group during the simulation
Wong et al. [30] 2018 Singapore	To understand the perceptions of CPR instructors towards using VR for HCPs’ CPR education: (1) current (2) features (3) the potential role Cross-sectional study Basic CPR ND ND	Convenience sampling 30 CPR instructors (5 physicians and 25 nurses) Age: 40 (SD 7.1) years Female: 25 Experience as CPR instructors: 7 (SD 3.8) years Academic hospital	This is a VR simulation which focuses on CPR and AED procedural training, which involves familiarizing oneself with the CPR steps and mastering their sequence Skills were performed by the avatar, and not the user The CPR + AED VR simulation uses Steam VR technology and is deployed on HTC VIVE, with sensors detecting the VR headset and controllers tracking users’ location and input Study phases were: - Questionnaire A (10 min), focused on perceptions towards current HCPs’ CPR education - Tutorial and Simulation modes of the CPR + AED VR Simulation (15 min) - Questionnaire B (10 min), about opinions on VR use for HCPs’ CPR education	ND	Instructors were surveyed before and after interacting with a CPR VR simulation Responses were analysed using interpretative thematic analysis The questionnaires were not validated Questionnaire A included 4 questions: 1 5-point Likert scale and 3 open questions Questionnaire B included 3 open questions	CPR instructors perceived current health professionals’ CPR education as limited due to not ideal test preparation (resources, practice, motivation, and frame of mind) and performance (technique, procedure sequence, landmark identification) They perceived fidelity, engagement, resource conservation and memory enhancement as features of VR ideal for HCPs’ CPR education VR was viewed by CPR instructors as having potential as a blended learning tool, targeting both ‘novice’ and ‘experienced’ HCPs
***Neonatal***						
Chang et al. [31] 2021 Los Angeles, USA	To describe VR development considerations from a conceptual lens, exploring why VR is an ideal modality to address resuscitation leadership skills To detail the collaborations and steps toward the first pilot product To provide data on the evaluation of VR simulations through the context of stress inoculation and the training of novice learners in paediatric resuscitation Randomized trial (pilot) Advanced CPR ND Began in 2016	Convenience sampling 34 physicians: 15 paediatric residents (novices) and 19 paediatric emergency medicine attending/fellow (experts) Age: ND Female: ND ND	VR scenarios were developed using the Unity 3D engine for use on the Oculus Rift Touch The software engine was similar to that used for entertainment games; it would be played on consoles, PCs, and mobile devices During the simulation, the participants saw the nurse and the respiratory therapist represented by avatars Two common paediatric resuscitation scenarios were represented: seizure and anaphylaxis Drop-down menus and suspended text in the air would severely impact the psychological fidelity of the scenario: they were eliminated and relied on all available equipment and medications on top of a cart as a form of multiple choice All participants were oriented to the VR hardware and then completed the tutorial and both scenarios in randomized order	Novices vs experts	The times taken to complete the tasks of each simulated scene were recorded in sec During VR performance, data for time-to-critical actions and stress physiology markers (heart rate, salivary cortisol) were collected The study occurred between 10 a.m. and 1 p.m	Attendees/fellows took longer to complete the tutorial than residents (172, 160–215 s vs 161, 158–163 s, p = 0.04) Performance data (e.g., oxygen masks, intravenous access, adrenaline) in the scenarios did not differ substantially in the beginning of between groups The end of the scenarios saw a few performance differences In the seizure scenarios, the attending/fellow group ordered fewer lorazepam doses (2, 2–4) before moving onto another antiepileptic when compared with resident groups (4, 3–6 doses, p = 0.003) In the anaphylaxis scenarios, time-to-cricothyrotomy in the attending/fellow group were much faster compared with the resident group (37, 12.75–44 s vs 86.5, 55.5–129.5 s, p = 0.02) The resident group had a slightly higher HR of 85.9 (78–93.7) compared to attending/fellow group, with 78.9 (71.2–87.4) beats per minute (p = 0.035) For salivary cortisol levels, the attending/fellow group had a 0.12 (IQR 0.09–0.17) μg/dL, whereas residents 0.19 (IQR 0.12–0.33) μg/dL The higher levels in residents were also statistically significant (+ 0.07 μg/dL, p = 0.001)
Ezenwa et al. [32] 2022 Lagos, Nigeria, and Busia, Kenya	To explore the feasibility and educational efficacy of using mobile VR for the precourse preparation of HCPs in neonatal resuscitation training Secondary analysis of a multicentre randomized controlled trial Basic CPR Training (precourse preparation) ND	Randomization for treatment or control groups 179 nurses and midwives (VR group: n = 91; control group: n = 88) who had not received training in HBB within the past 1 year Age: 37 (SD 9) years Female: 162 (90.5%) Obstetrics and newborn care units 20 secondary and tertiary health-care facilities	The eHBB VR simulations consisted of 3 brief interactive 3D simulation scenarios that represented a newborn requiring routine care, some resuscitation, or prolonged resuscitation via positive pressure ventilation The simulations were accessed using a low-cost VR headset and the eHBB virtual simulation app installed on mobile phones The VR scenarios were completed within 3 to 5 min An advanced manikin was used for the standardized collection of data on BMV skills A minimum of 20 min was given for the participants to familiarize themselves with their study group materials	Participants were randomized to receive the digitized HBB Provider’s Guide (VR group) intervention or the digitized HBB Provider’s Guide–only intervention (control group) before a standard in-person HBB course	Precourse knowledge and skills assessments with - the HBB knowledge check multiple-choice questionnaire - the BMV skills check - the OSCE checklist	The overall performance scores on the knowledge check (p = 0.29), bag and mask ventilation skills check (p = 0.34), and OSCE checklist (p = 0.43) were similar between groups During the OSCE the VR group performed better on the critical step of positioning the head and clearing the airway (VR group: 77/90, 86%; control group: 57/88, 65%; p = 0.002) The median percentage of ventilations that were performed via head tilt, as recorded by the manikin, was also higher in the VR group (75%, IQR 9%–98%) than in the control group (62%, IQR 13%–97%), (p = 0.35). Participants in the control group performed better on identifying a helper and reviewing the emergency plan step (VR group: 7/90, 8%; control group: 16/88, 18%; p = 0.045) and the washing-hands step (VR group: 20/90, 22%; control group: 32/88, 36%; p = 0.048)
Umoren et al. [33] 2021 Lagos, Nigeria, and Busia, Kenya	To assess the impact of mobile VR simulations using eHBB or video for the maintenance of neonatal resuscitation skills in HCPs in resource-scarce settings Multicentre, randomized controlled trial Basic CPR Retraining December 2018– August 2019, with 6-month follow-up	Three-arm randomization (computer-generated algorithm) Participants were enrolled and assigned a study ID before the HBB course by local coordinators (sample size 83 subjects/Group) Excluded those having attended neonatal resuscitation training in the year prior to the study 274 nurses and midwives Age: 38 (9) years Female: 250 (91.2%) 92.5% of participants owned a mobile phone Labour and delivery, operating room and newborn care units from 20 health-care facilities	A 30-min orientation was provided on the use of the app Participants had access to a digitized HBB provider manual through the app The VR group in addition accessed the eHBB VR simulations consisting of three interactive three-dimensional simulation scenarios representing care of a newborn requiring routine care, some resuscitation and prolonged resuscitation with positive pressure ventilation None of the interventions required Internet for use The HBB provider course (second edition) was taught by study HBB master trainers as 1-day, 8-h long sessions	Three study groups: VR (eHBB + digital guide), video (video + digital guide) or control (digital guide only) groups before an in-person HBB course	The HBB knowledge check and bag-and-mask ventilation skill check were conducted precourse and post-course along with the OSCE A checklist on preparation for delivery and initial steps of resuscitation. In addition, the post-course assessment included the OSCE B checklist on prolonged newborn resuscitation A demographic survey was also completed Following the course, participants were encouraged to use their assigned digital intervention weekly and to engage in standard bag-and-mask skills practice with a manikin at the HBB practice corner set up at their facility. Post-course assessments were repeated at 1, 3 and 6 months	Neonatal resuscitation skills pass rates were similar among the groups at 6-month follow-up for BMV skills check (VR 28%, video 25%, control 22%, p = 0.71), OSCE A (VR 76%, video 76%, control 72%, p = 0.78) and OSCE B (VR 62%, video 60%, control 49%, p = 0.18) In the immediate post-course, there was greater retention of BMV skills at 6 months in the VR group (− 15% VR, p = 0.10; − 21% video, p < 0.01, –27% control, p = 0.001) OSCE B pass rates in the VR group were higher at 3 months (+ 4%, p = 0.64) and 6 months (+ 3%, p = 0.74) and lower in the video (− 21% at 3 months, p < 0.001; − 14% at 6 months, p = 0.066) and control groups (− 7% at 3 months, p = 0.43; − 14% at 6 months, p = 0.10) On follow-up survey, 95% (n = 65) in the VR group and 98% (n = 82) in the video group would use their assigned intervention again

*ACLS* Advanced Cardiac Life Support, *AED* Automatic External Defibrillator, *AHA* American Heart Association, *AI* Artificial Intelligence, *ALS* Advanced Life Support, *BLS* Basic Life Support, *BMV* bag and mask ventilation, *bpm* beats per minutes, *CA* cardiac arrest, *CC* chest compressions, *CPR* Cardio-Pulmonary Resuscitation, *CPVR-sim* Cardio Pulmonary Virtual Reality Simulation, *CSU* cardiac surgical unit, *eHBB* electronic helping babies breathe, *FB* feedback, *HBB* helping babies breathe, *HCPs* health-care professionals, *HFS* high-fidelity simulation, *HR* heart rate, *ICU* intensive care unit, *IQR* inter quartile range, *mini-VREM* mini-virtual reality, *NASA-TLX* National Aeronautics and Space Administration Task Load Index, *ND* not defined, *NFB* non-feedback, *OSCE* Objective Structured Clinical Examination, *P* probability, PC personal computer, *PEA* pulseless electrical activity, *RN* registered nurse, *SBAR* situation, background, assessment, recommendation, *SD* Standard Deviation, *USA* United States of America, *VF* ventricular fibrillation, *VR* virtual reality, *VT* ventricular tachycardia, *XR* extended reality

Six were primarily aiming to investigate the efficacy of VR CPR training [18, 23, 25, 29, 31, 33], four its feasibility [22, 28, 29, 32], three its utility [19, 24, 30], and two its usability [20, 26]. To reach these aims, six were RCTs [22, 25, 27, 29, 31, 33], six were cross-sectional studies [19, 20, 24, 26, 28, 30]_,_ and three quasi experimental [18, 23, 32]_._

Basic CPR maneuvers were the target of eight studies [19, 22, 23, 26, 29, 30, 32, 33], while the remaining considered advanced CPR maneuvers [18, 20, 24, 25, 27, 28, 31]. Moreover, five studies focused on the initial CPR training [20, 22, 25, 27, 32], two the retraining [24, 33], while the remainder did not specify [18, 19, 23, 26, 28–31]. Studies were conducted mainly in hospitals [19, 22, 28–30, 32, 33], training centers [20, 23–25], and universities [18, 27]; two studies did not specify the setting [26, 31].

Regarding the targeted patients, population for which the training was intended included in 12 studies adults [18–20, 22–30]) while in three neonates [31–33]; no pediatric patients were included. In the neonatal field participants were only HCPs, with 274 nurses and midwives [33], and 34 physicians [31]. Differently, studies concerning adult CPR included only physicians and sub-specialty, specific participants ranging from 25 anesthesiology residents to 148 clinicians [24, 25]; other adult studies included a mixed of HCPs, as for example 40 physicians and nurses [18], or 30 CPR instructors [30]. Some studies were focused on a limited number of professionals (e.g., three nursing assistants and four nurses [22]) to 80 participants including nurses, physicians, engineers, students and researchers [29]. The age of participants ranged from 19 to 57 years and studies included primarily females (e.g., 93.2% [25]); however, five studies did not specify participants’ sex or age [20, 22, 23, 26, 31].

At the overall level, the data collection duration lasted from two years with an additional six-month follow-up to a single day [27, 33]. The remaining studies collected data over two and over three months [18, 22, 29]; however, in 11 studies the duration was not specified.

The methodological quality of the studies (Supplementary tables 2, 3 and 4) was overall fair; in the six cross-sectional studies confounding factors and strategies to deal with them were not identified [19, 20, 24, 26, 28, 30]; in five RCTs the allocation to treatment groups and the outcome assessors were not blinded [25, 27, 29, 31, 32]; in two quasi-experimental studies the control group was not identified [18, 22].

### VR CPR intervention and comparison training methods

#### Intervention group

Almost all studies carried out the VR training using virtual 3D scenarios with the aid of additional accessories, such as a viewer [19]. Some studies also employed a high-fidelity manikin connected to a computer [19, 20, 23, 26, 29, 32], and compared the use of a very persuasive scenario with many components (visual aids, live instructions, and text messages) and a less persuasive one (only task completion messages at the end of these) [20]. In some studies, instructors were allowed to independently modify scenarios in real time [19, 24, 28].

Five studies showed an initial tutorial regarding the VR simulation [18, 22, 26, 27, 31]; which varied from a 20-s presentation to 20 min with an AI instructor [22, 26]. The total duration of the training session was usually one hour [18, 27], but the VR exposure time ranged from three or five minutes per scenario to approximately 30 min [20, 32].

#### Comparison group

The training methods considered as comparators were mainly the traditional CPR using a full-body or half-torso low-fidelity manikin [25, 27, 29], or a high-fidelity manikin [24]; or two different versions of the VR simulator (with and without a persuasive features) [20]. In the other two studies the comparators were not specified [23, 30]. Moreover, a pre-post comparison was performed with the same group [18, 22]; while two groups, with different qualifications and experiences, were compared within the same VR simulation [28, 31].

### VR CPR outcomes

No studies investigated patient’s outcomes whereas those studies reporting HCPs outcomes were mainly based on a pre-post [18, 22], or only post- questionnaires [19, 20, 24, 26–28, 30, 32], through checklists filled in by observers [22, 25, 33], or specific sensors and software (e.g., feedback technology [29]).

### Outcomes at the HCP level

#### HCP experience

At the HCP experience level, VR scenarios were considered realistic (e.g., 80% [18]; 97% [19]). Moreover, around 72% of participants perceived the environment as engaging and facilitating the memorization of the procedures; 86% considered the VR simulations a useful training and agreed to recommend it to other colleagues [27]. On the other hand, the high-fidelity simulation group reported similar satisfaction and utilization scores, but significantly better scores regarding the provided feedback (99%, Interquartile Range [IQR] 89–100) as compared to the VR group (79%, IQR 71–88, p < 0.01) [24]. Using VR has led to greater commitment and increased knowledge acquired; furthermore, the gaming approach was perceived positively [30]. However, some disadvantages emerged in using VR scenarios, including limited decision-making freedom, the lack of the possibility to build a team, as well as the absence of a real psychological pressure and a frenetic environment, as lived in the reality [28]. In this regard, the level of stress during VR simulations among residents showed higher salivary cortisol levels than consultants (+ 0.07 μg/dL, 95%, CI = 0.03–0.12, p = 0.001) [31]. During the same VR simulations, heart rates also differed between residents and consultants, with higher values for the former (85.9 hearth rate per minute, range 78–93.7) compared to the latter (78.9 hearth rate per minute, range 71.2–87.4) [31].

#### HCP knowledge

At the HCP knowledge level, studies comparing the number of correct questions through a pre–post test showed a statistically significant improvement [18, 22, 33]. For example, the number of correct answers per HCP participants increased on average by 4.8 (95%, CI 3.4–6.2, p < 0.001) after the virtual simulation [18].

#### HCP performance

Performance measured as the degree of confidence with the automated external defibrillator (e.g. improvement in the time of positioning the pads, in rhythm analysis and defibrillation) and participation in a group simulation, was higher (+ 28.6% both) after virtual simulation [22]. In contrast, 43% of participants after the traditional training were able to administer stacked shocks within one minute, whereas none of the participants in the VR group could so; moreover, the target sternotomy time was achieved in 100% of procedures in the control group and in 83% in the VR group [27]. However, in the VR group the total number of errors was lower compared to the control group (11 vs 15) [27]. The more previous training in CPR possessed by a participant the greater the percentage of correct compressions obtained in a VR CPR session (F-Ratio 14.95; p < 0.001) [23]. A total of 88% of participants using VR achieved a correct compression rate and 65% a positive result in the depth of compression applied to the manikin [19]. Better retention of skills in bag and mask ventilation at six months (-15% VR group, p = 0.10; -21% video group, p < 0.01; -27% control group, p = 0.001) was documented [33].

An improvement was also found when comparing the pre–post performances of the low-fidelity manikin group (p = 0.017 for pulseless electrical activity, PEA, p = 0.01 for ventricular fibrillation/ventricular tachycardia, VF/VT) and the persuasive VR group (p = 0.02 for PEA, p = 0.048 for VF/VT) but not for the minimally persuasive VR group (p = 0.45 for PEA, p = 0.46 for VF/VT) [25]. Different, participants in the group with the less persuasive scenario reported a perception of greater usability (p = 0.0944) and ease in interfacing with the simulation compared to the very persuasive scenario (p = 0.0813) [20]; however, the results were not statistically significant. The group with live feedback on the quality of chest compressions achieved better performance in both frequency and depth compared to the non-feedback group (35.78% vs 7.27%, p < 0.001) [29]. It was also seen that the chest compressions rate of the more experts was not influenced by the feedback, while the performances of the novices improved with the use of the feedback (112.07, SD 3.70 vs 98.39, SD 29.23, p = 0.001) [29].

### Outcomes at the health-care system level

#### Economic Impact

At the organizational level, four studies mentioned the cost-saving opportunities that VR could have in the future, despite the initial expense for the purchase of software (as licenses) and materials (as computers, sensors) [18, 24, 28, 31]. However, only one calculated the economic impact of using VR compared to the traditional method, highlighting a significant saving of 83% for a single learner for four training sessions (132.29 dollars for VR versus 772.00 dollars for the traditional method) in terms of human resources, classrooms as well as time spent in travelling [24].

## Discussion

### Study characteristics

The research in the field of VR CPR has been established in the last ten years, with 15 studies, including 1,035 total participants and conducted in almost all continents, suggesting that these educational strategies can be ubiquitarian and considered also by lower-income countries. As documented in a recent systematic review, VR CPR training among adult laypersons has instead been investigated by only six studies involving a total of 731 participants between 2017 and 2021 [10].

Measuring the effectiveness, the feasibility and the utility of VR CPR has been the main intents of studies available; therefore, this research field seems to be focused around two main lines. The first is aimed at developing and introducing VR into CPR training, and the second in measuring its outcomes, both in basic and advanced CPR skills, mainly with regards to adult patients, involving HCPs in hospital settings. This substantial differences in research approaches, divided between educational impact [31], and those of digital devices design, development and piloting [20], may explain some missing elements that have emerged in the study methodology quality assessment as: study duration, mainly short or not reported; limited sample size, from a few HCPs to a maximum of 274; lack of description of the control group, or no control group established (four studies); and the poor description of the professional background, baseline competences and/or participants’ experiences. The lack of description of the HCPs expertise, should be considered as an important lack in assessing whether the outcomes are purely associated to the VR CPR or also to prior experience. Overall, studies available seem to express a research field in its early stage. Guidelines based on broad consensus regarding the elements to document in future studies to harmonize their reporting and ensure that some essential data are described (e.g., VR CPR as a refresher or first training), by differentiating those aimed at piloting the digital technologies and those at measuring their effectiveness, may increase transparency, comparability and the accumulation of the evidence produced.

### Intervention and comparison group training methods

Five main considerations may be derived from VR devices and training methods documented to date in the available literature.

First, according to the extended reality recently conceptualized in this field [2], twelve studies [18–20, 23–25, 27, 28, 30–33] were based on VR, two on augmented reality [26, 29], while one used the term virtual simulation [22]. Therefore, studies available mainly used VR and augmented reality-based CPR training.

Second, while some studies have been based on relatively simple technologies [20, 29], others have implied several and complex technologies, from software created for 3D gaming to sensors connected to a computer [18, 24, 30]. On the one hand, this great heterogeneity suggests an ample creativity and innovation; on the other, it prevents comparison and conclusions regarding the outcomes.

Third, interactivity as the possible way to provide (or not) real-time feedback on participants’ performance (e.g., depth and frequency of chest compressions) and the likelihood of changing the scenario suggest that some studies embraced the main characteristics of VR, i.e. that it includes a totally immersive environment in which the person is completely isolated from the external setting and transported into a parallel reality, reproduced with the use of additional devices, such as visors or sensory gloves [19, 31].

Fourth, in all studies, the sections are short in duration, sometimes providing brief tutorials to familiarize participants with the tasks; in all cases, from the initiation to the end of the section, the CPR training seems to save time, and this may benefit the HCPs, the health-care organization and the continuing education agencies to address other learning needs. Regarding the comparisons, a wide range of training methods have been used: alongside efforts in standardizing the description of the comparator used in the studies, comparing the effectiveness of VR CPR with the most traditional training method used in daily practice may help in accumulating evidence in this field.

### VR CPR outcomes

Different outcomes have been measured to date, employing several measures both subjective (e.g., self-reported participants’ satisfaction [24]) and objective (e.g., sensor for automatic detection of participants’ performance [19]), preventing the production of a cumulative synthesis of the evidence available. First, the substantial infancy of this research field may have prevented any evaluation on patient outcomes, requiring complex study designs and long follow-ups. Also, in the review concerning lay people, a few studies have investigated the association of the educational interventions with patients’ outcomes after cardiac arrest, both in the short and long term [10]. However, given that the main aims of the CPR training, regardless of the methods, is to improve health outcomes, this lack should be addressed in the future to assess the VR role in this training field.

Second, outcomes most evaluated are those at the HCP level. With this regards, Kirkpatrick’s framework has established the evaluation system of HCP educational programs as categorized into four levels, from the first and easier to assess (reactions) to the following and more complex (learning, behavioral and results) [34]. In the context of VR CPR training, the first have been documented in 13 studies [18–20, 22–27, 29, 31–33], reporting a good level of usability, appreciation, and satisfaction. One study underlined that the simulation failed to reproduce the characteristics of the real emergency scenario [28].

Learning outcomes, the second level of the Kirkpatrick’s framework [34], have been measured in all studies. Knowledge has improved, along with some evidence regarding better memory retainment of the acquired practices [18]. With the traditional CPR education, the decline in theoretical notions and practical skills occurs just two months after the end of the course, reaching a peak at six months [35]; in contrast, VR training has shown that knowledge and skills at one, three, and six months remain higher [33]. Moreover, some better performances have also been reported in terms of the decreased time needed to assess the heart rhythm and use of the defibrillator, which can be particularly useful in rare conditions, such as cardiac arrest in children [36]. Notably, two out of three studies concerning neonatal emergencies were conducted in Africa [32, 33], where the neonatal mortality rate is extremely high suggesting that VR methods of training may have a potential role in some conditions characterized by the rare occurrence of the phenomenon in the real world, issues regarding geographic accessibility, and underdeveloped economic conditions [37].

No studies investigated behavioral modifications [34], such as the extent to which the trainees apply the learning and change their behavior in real practice, immediately or months after the training. Conducting prospective studies monitoring the behavioral modification of participant HCPs after VR CPR training over time may be challenging, time-consuming and influenced by several confounding factors (e.g., having attended other training initiatives [38]). Most training interventions are evaluated only in terms of theoretical-practical knowledge achieved immediately at the end, and not in terms of changes in the medium or long term [39, 40]. In addition, only a few health-care facilities and universities use VR, and this may limit the likelihood of conducting such evaluations [41].

Alongside the lacks in behaviors modification, VR CPR training has been subjected to a little scrutiny regarding its capacity to develop decision making and team-building skills (e.g., team training and interaction with the team) [28]. Both skills have been described as fundamental in the CPR practice [41], and this limitation might affect the real-world applicability of VR in this field. Involving more participants within the same VR scenario and promoting their interaction over that between the HCPs and the VR software may be useful [28]; reproducing the chaotic environment typical lived during the emergency situations, may also increase the reality of the VR scenarios and promote the transferability of the competences gained in the real world.

Concerning the impact of the results on the health-care organization, no studies have assessed this association with VR CPR training also in this case likely due to the complexity of these investigations [42, 43]. Despite some studies reported cost-saving opportunities [18, 28, 31], only one calculated the economic impact [24]. Understanding whether VR can have benefits in terms of efficiency is essential to promote and convince of the need of an initial investment that may have important long-term cost-saving effects [44]. Producing more evidence may also inform the long-term sustainability of investments in these new technologies.

### Limitations

This systematic review has several limitations. Despite the attempt to conduct an accurate systematic review by registering in advance the protocol, and strictly following the PRISMA guidelines [11], selection biases may have occurred. Moreover, to provide a broader picture on all studies available all retrieved articles have been included despite their methodological quality. In addition, the background of researchers involved in the analysis of the studies, although experts in CPR and research methodologies, may have influenced the data extraction by missing some aspects regarding the technologies used.

## Conclusions

The research in the field of VR CPR training is recent and conducted in several countries, mainly focused around two aims: developing the VR technologies and methods of training; and measuring the VR CPR effectiveness or practical implementation. Studies available concern mainly adult CPR training in hospital settings, with mixed voluntary HCPs; they are not-always specified as training or retraining and limited in the sample size. Outcomes have been assessed mainly at the HCP level, investigating reactions, and effects on knowledge and performance.

At the overall level, the evidence available regarding VR CPR training is promising: however, robust studies are required to overcome the methodological gaps in this research field. Guidelines establishing the essential data that should be communicated while reporting studies are recommended. Moreover, the effectiveness of VR CPR training research on clinical outcomes (by considering also pediatric patients) should be investigated; in addition, describing changes in the performances and behavior as manifested in the management of real-world cases after having received the VR CPR training is a priority. In this context, designing more complex studies by involving several HCPs in the same scenario, connected remotely, and stimulating interactions and strategies to also investigate their decision-making processes, may be important. All these evaluations may expand the evidence available and inform decision-makers regarding long-term investments and sustainability of the VR CPR training.

## Supplementary Information

Below is the link to the electronic supplementary material.

Supplementary file1 (DOCX 40 KB)

## Data Availability

Not applicable.
