# Supplementary material for: Virtual Reality for Cardiopulmonary Resuscitation Healthcare Professionals Training: A Systematic Review
Source: J Med Syst. 2024 May 15;48(1):50. doi: 10.1007/s10916-024-02063-1 (PMC11096216; doi:10.1007/s10916-024-02063-1)
Supplement: Supplementary file 1 — Supplementary file1 (DOCX 40 KB) [file 10916_2024_2063_MOESM1_ESM.docx]

Journal of Medical Systems

Supplementary material

**Virtual reality for cardiopulmonary resuscitation**

**healthcare professionals training: a systematic review**

Trevi R, Chiappinotto S, Palese A, Galazzi A.

**Supplementary Table 1.** Search strings

| **Database** | **Data** | **Query** |
| --- | --- | --- |
| PubMed | On 7 June 2023 | (Healthcare professional [TIAB]) AND (virtual reality [TIAB]) AND (resuscitation [TIAB]) |
| Scopus | On 9 June 2023 | (TITLE-ABS-KEY (virtual AND reality) AND TITLE-ABS-KEY (resuscitation) OR TITLE-ABS-KEY (cardiopulmonary AND resuscitation)) AND (LIMIT-TO (SUBJAREA,"MEDI”) OR LIMIT-TO (SUBJAREA,"COMP”) OR LIMIT-TO (SUBJAREA,"ENGI”) OR LIMIT-TO (SUBJAREA,"NURS”) OR LIMIT-TO (SUBJAREA,"HEAL”) |
| Cochrane | On 9 June 2023 | "Virtual reality" in All Text AND "resuscitation" in All Text |
| CINAHL | On 9 June 2023 | ("virtual reality"[Title/Abstract]) AND ("resuscitation"[Title/Abstract]) |

**Legend:** CINAHL, Cumulative Index to Nursing and Allied Health Literature.

**Supplementary Table 2.** Quality appraisal of the included studies according to the Joanna Briggs Institute methodology, tool for quasi-experimental studies [16]

| **Included Studies** | **Item 1**. Is it clear in the study what is the ‘cause’ and what is the ‘effect’ (i.e., there is no confusion about which variable comes first)? | | | **Item 2**. Were the participants included in any comparisons similar? | | | **Item 3**. Were the participants included in any comparisons receiving similar treatment/care, other than the exposure or intervention of interest? | | | **Item 4**. Was there a control group? | | | **Item 5**. Were there multiple measurements of the outcome both pre and post the intervention/ exposure? | | | **Item 6**. Was follow up complete and if not, were differences between groups in terms of their follow up adequately described and analyzed? | | | **Item 7**. Were the outcomes of participants included in any comparisons measured in the same way? | | | **Item 8**. Were outcomes measured in a reliable way? | | | **Item 9.** Was appropriate statistical analysis used? | | |
| --- | --- | --- | --- | --- | --- | --- | --- | --- | --- | --- | --- | --- | --- | --- | --- | --- | --- | --- | --- | --- | --- | --- | --- | --- | --- | --- | --- |
|  | **R^1^** | **R^2^** | **C** | **R^1^** | **R^2^** | **C** | **R^1^** | **R^2^** | **C** | **R^1^** | **R^2^** | **C** | **R^1^** | **R^2^** | **C** | **R^1^** | **R^2^** | **C** | **R^1^** | **R^2^** | **C** | **R^1^** | **R^2^** | **C** | **R^1^** | **R^2^** | **C** |
| Brzozowski et al., 2021 [22] | NA | Y | **Y** | N | N | **Y** | N | U | **Y** | N | N | **N** | Y | Y | **Y** | NA | N | **N** | N | Y | **Y** | Y | Y | **Y** | Y | Y | **Y** |
| Buttussi et al., 2013 [18] | Y | Y | **Y** | Y | U | **Y** | Y | U | **Y** | N | N | **N** | Y | Y | **Y** | Y | N | **Y** | Y | Y | **Y** | Y | N | **N** | Y | Y | **Y** |
| García Fierros et al., 2021 [23] | Y | Y | **Y** | N | U | **N** | Y | U | **Y** | N | U | **Y** | N | N | **N** | Y | N | **Y** | Y | Y | **Y** | Y | U | **Y** | Y | Y | **Y** |

**Legend:** Y: yes; N: no; U: unclear; NA: not applicable; C: Consensus; R^1^: Rater 1; R^2^: Rater 2

**Supplementary Table 3.** Quality appraisal of the included studies according to the Joanna Briggs Institute methodology, tool for cross-sectional studies [17]

| **Included Studies** | **Item 1**. Were the criteria for inclusion in the sample clearly defined? | | | **Item 2**. Were the study subjects and the setting described in detail? | | | **Item 3**. Was the exposure measured in a valid and reliable way? | | | **Item 4**. Were objective, standard criteria used for measurement of the condition? | | | **Item 5**. Were confounding factors identified? | | | **Item 6**. Were strategies to deal with confounding factors stated? | | | **Item 7**. Were the outcomes measured in a valid and reliable way? | | | **Item 8**. Was appropriate statistical analysis used? | | |
| --- | --- | --- | --- | --- | --- | --- | --- | --- | --- | --- | --- | --- | --- | --- | --- | --- | --- | --- | --- | --- | --- | --- | --- | --- |
|  | **R^1^** | **R^2^** | **C** | **R^1^** | **R^2^** | **C** | **R^1^** | **R^2^** | **C** | **R^1^** | **R^2^** | **C** | **R^1^** | **R^2^** | **C** | **R^1^** | **R^2^** | **C** | **R^1^** | **R^2^** | **C** | **R^1^** | **R^2^** | **C** |
| Katz et al., 2020 [24] | Y | Y | **Y** | Y | Y | **Y** | Y | Y | **Y** | Y | N | **Y** | N | N | **N** | N | N | **N** | Y | Y | **Y** | Y | Y | **Y** |
| Lee et al., 2022 [26] | Y | Y | **Y** | Y | U | **U** | Y | Y | **Y** | Y | N | **N** | N | N | **N** | N | N | **N** | Y | N | **N** | NA | Y | **N** |
| Rodríguez-Matesanz et al., 2022 [19] | N | N | **N** | Y | Y | **Y** | Y | Y | **Y** | N | N | **N** | N | N | **N** | N | N | **N** | N | N | **N** | N | U | **N** |
| Sadeghi et al., 2022 [28] | Y | N | **N** | Y | Y | **Y** | Y | Y | **Y** | Y | N | **N** | N | N | **N** | N | N | **N** | Y | N | **N** | Y | Y | **Y** |
| Vankipuran et al., 2014 [20] | Y | Y | **Y** | N | N | **N** | Y | Y | **Y** | U | N | **Y** | N | N | **N** | N | N | **N** | N | N | **N** | N | N | **N** |
| Wong et al., 2018 [30] | Y | U | **Y** | Y | Y | **Y** | Y | Y | **Y** | N | N | **N** | N | N | **N** | N | N | **N** | Y | N | **N** | NA | U | **U** |

**Legend:** Y: yes; N: no; U: unclear; NA: not applicable; C: Consensus; R^1^: Rater 1; R^2^: Rater 2

**Supplementary Table 4.** Quality appraisal of the included studies according to the Joanna Briggs Institute methodology, tool for Randomized Controlled Trials [15]

| **Included Studies** | **Item 1**. Was true randomization used for assignment of participants to treatment groups? | | | **Item 2**. Was allocation to treatment groups concealed? | | | **Item 3**. Were treatment groups similar at the baseline? | | | **Item 4**. Were participants blind to treatment assignment? | | | **Item 5**. Were those delivering the treatment blind to treatment assignment? | | | **Item 6**. Were treatment groups treated identically other than the intervention of interest? | | | **Item 7**. Were outcome assessors blind to treatment assignment? (Outcome 1, 2, …) | | | **Item 8**. Were outcomes measured in the same way for treatment groups? (Outcome 1, 2, …) | | | **Item 9.** Were outcomes measured in a reliable way (Outcome 1, 2, …) | | |
| --- | --- | --- | --- | --- | --- | --- | --- | --- | --- | --- | --- | --- | --- | --- | --- | --- | --- | --- | --- | --- | --- | --- | --- | --- | --- | --- | --- |
|  | **R^1^** | **R^2^** | **C** | **R^1^** | **R^2^** | **C** | **R^1^** | **R^2^** | **C** | **R^1^** | **R^2^** | **C** | **R^1^** | **R^2^** | **C** | **R^1^** | **R^2^** | **C** | **R^1^** | **R^2^** | **C** | **R^1^** | **R^2^** | **C** | **R^1^** | **R^2^** | **C** |
| Chang et al., 2021 [31] | N | U | **N** | N | U | **N** | N | N | **N** | N | N | **N** | N | U | **N** | Y | Y | **Y** | 1: N  2: N | 1: U  2: U | **1: N**  **2: N** | 1: Y  2: Y | 1: Y  2: Y | **1: Y**  **2: Y** | 1: Y  2: Y | 1: Y  2: Y | **1: Y**  **2: Y** |
| Ezewna et al., 2022 [32] | Y | U | **U** | N | U | **N** | Y | Y | **Y** | N | N | **N** | N | N | **N** | N | Y | **Y** | 1: N | 1: N  2: N | **1: N** | 1: Y | 1: Y  2: Y | **1: Y** | 1: Y | 1: Y  2: Y | **1: Y** |
| Khanal et al., 2014 [25] | Y | U | **U** | N | U | **N** | Y | U | **Y** | N | U | **N** | N | U | **N** | Y | U | **Y** | 1: N | 1: U | **1: N** | 1: Y | 1: Y | **1: Y** | 1: Y | 1: N | **1: Y** |
| Peek et al., 2023 [27] | Y | Y | **Y** | N | Y | **N** | Y | Y | **Y** | N | N | **N** | N | N | **N** | N | Y | **Y** | 1: N  2: N | 1: Y  2: Y  3: Y  4: Y | **1: Y**  **2: Y**  **3: Y**  **4: Y** | 1: Y  2: Y | 1: Y  2: Y  3: Y  4: Y | **1: Y**  **2: Y**  **3: Y**  **4: Y** | 1: Y  2: Y | 1: Y  2: Y  3: Y  4: Y | **1: Y**  **2: Y**  **3: Y**  **4: Y** |
| Semeraro et al., 2013 [29] | Y | U | **Y** | N | U | **N** | N | U | **N** | N | Y | **N** | N | Y | **N** | N | Y | **Y** | 1: N | 1: U | **1: N**  **2: N** | 1: Y | 1: Y | **1: Y**  **2: Y** | 1: Y | 1: N | **1: Y**  **2: Y** |
| Umoren et al., 2021 [33] | Y | Y | **Y** | Y | Y | **Y** | Y | Y | **Y** | N | U | **Y** | N | U | **N** | Y | Y | **Y** | 1: N | 1: N  2: N  3: N | **1: Y**  **2: Y**  **3: Y** | 1: Y | 1: Y  2: Y  3: Y | **1: Y**  **2: Y**  **3: Y** | 1: Y | 1: Y  2: Y  3: Y | **1: Y**  **2: Y**  **3: Y** |

**Supplementary Table 4 (continued)**

| **Included Studies** | **Item 10**. Was follow up complete and if not, were differences between groups in terms of their follow up adequately described and analysed? (Outcome 1: result 1, 2, …; Outcome 2: result 1, 2, …) | | | **Item 11**. Were participants analysed in the groups to which they were randomized? (Outcome 1: result 1, 2, …; Outcome 2: result 1, 2, …) | | | **Item 12**. Was appropriate statistical analysis used? (Outcome 1: result 1, 2, …; Outcome 2: result 1, 2, …) | | | **Item 13**. Was the trial design appropriate and any deviations from the standard RCT design (individual randomization, parallel groups) accounted for in the conduct and analysis of the trial? | | |
| --- | --- | --- | --- | --- | --- | --- | --- | --- | --- | --- | --- | --- |
|  | **R^1^** | **R^2^** | **C** | **R^1^** | **R^2^** | **C** | **R^1^** | **R^2^** | **C** | **R^1^** | **R^2^** | **C** |
| Chang et al., 2021 [31] | 1: Y, Y  2: Y, Y | 1: U  2: U | **1: Y**  **2: Y** | 1: Y  2: Y | 1: N  2: N | **1: Y**  **2: Y** | 1: Y  2: Y | 1: Y  2: Y | **1: Y**  **2: Y** | N | N | **N** |
| Ezewna et al., 2022 [32] | 1: Y | 1: Y  2: Y | **1: Y** | 1: Y | 1: N  1: N | **1: Y** | 1: Y | 1: Y  2: Y | **1: Y** | Y | U | **Y** |
| Khanal et al., 2014 [25] | 1: Y | 1: N | **1: Y** | 1: Y | 1: N | **1: Y** | 1: Y | 1: N | **1: Y** | Y | N | **Y** |
| Peek et al., 2023 [27] | 1: Y  2: Y | 1: Y  2: Y  3: Y  4: Y | **1: Y**  **2: Y**  **3: Y**  **4: Y** | 1: Y  2: Y | 1: U  2: U  3: U  4: U | **1: N**  **2: N**  **3: N**  **4: N** | 1: Y  2: Y | 1: Y  2: Y  3: Y  4: Y | **1: Y**  **2: Y**  **3: Y**  **4: Y** | Y | Y | **Y** |
| Semeraro et al., 2013 [29] | 1: Y | 1: Y | **1: Y**  **2: Y** | 1: Y | 1: N | **1: Y**  **2: Y** | 1: Y | 1: N | **1: Y**  **2: Y** | Y | U | **Y** |
| Umoren et al., 2021 [33] | 1: Y | 1: Y  2: Y  3: Y | **1: Y**  **2: Y**  **3: Y** | 1: Y | 1: N  2: N  3: N | **1: Y**  **2: Y**  **3: Y** | 1: Y | 1: Y  2: Y  3: Y | **1: Y**  **2: Y**  **3: Y** | Y | Y | **Y** |

**Legend:** Y: yes; N: no; U: unclear; NA: not applicable; C: Consensus; R^1^: Rater 1; R^2^: Rater 2
